# Audit: Anticholinergic Burden in Patients Newly Referred to the Central Aberdeenshire CMHT (Feb 2024–Feb 2025)

**DOI:** 10.1192/bjo.2026.11416

**Published:** 2026-06-30

**Authors:** Theam Hou Lee, JunTat Tan

**Affiliations:** Royal Cornhill Hospital, Aberdeen, United Kingdom

## Abstract

**Aims:**

To quantify anticholinergic burden among older adults newly referred to the Central Aberdeenshire Community Mental Health Team (CMHT) between February 2024 and February 2025, and to evaluate whether clinicians documented interventions to review or mitigate anticholinergic medications when the burden was clinically significant.

**Methods:**

This quality improvement project reviewed all general practitioner referrals to the Central Aberdeenshire CMHT during the study period. Of 241 referrals, 30 deceased patients were excluded, leaving 211 patient records for analysis. Prescribed medications were reviewed and anticholinergic burden was calculated using both the German Anticholinergic Burden Score and the Anticholinergic Cognitive Burden (ACB) Scale; where discrepanciesoccurred, the higher score was used to prioritise patient safety. An ACB score ≥3 was considered clinically significant. For these patients, clinical records were examined to identify documented interventions, categorised as switching to less anticholinergic alternatives, suggesting a medical review, deprescribing, or dose adjustment/reduction.

**Results:**

Among the 211 patients (age range 61–101 years), 73% were prescribed at least one anticholinergic medication and 32.7% (N=69) had a clinically significant anticholinergic burden (ACB ≥3), with scores ranging from 0 to 11. Anticholinergic burden was strongly associated with polypharmacy: 63.9% of patients prescribed eight or more medications had an ACB ≥3, compared with 11.8% of those on fewer than five medications. High-burden medications most frequently prescribed included quetiapine, amitriptyline, and solifenacin.

Despite the prevalence of significant anticholinergic burden, 75.4% of affected patients had no documented intervention. Interventions included deprescribing (8.7%), dose adjustment or reduction (8.7%), suggesting a medical review (4.4%), and switching to a less anticholinergic alternative (2.9%).

**Conclusion:**

Clinically significant anticholinergic burden remains common among older adults referred to CMHT services and is closely linked to polypharmacy. However, documented clinical interventions have declined compared with previous local audits, indicating a widening gap between risk identification and active management. Embedding routine anticholinergic burden calculation into assessments, prompting systematic medication review for patients with ACB ≥3, and supporting clinicians with targeted education may improve patient safety. A re-audit is planned to evaluate the impact of these interventions.

